# Revealing Facts and Avoiding Biases: A Review of Several Common Problems in Statistical Analyses of Epidemiological Data

**DOI:** 10.3389/fpubh.2016.00207

**Published:** 2016-10-07

**Authors:** Lihan Yan, Yongmin Sun, Michael R. Boivin, Paul O. Kwon, Yuanzhang Li

**Affiliations:** ^1^Office of Biostatistics and Epidemiology, Center for Biologics Evaluation and Research, U.S. Food and Drug Administration, Silver Spring, MD, USA; ^2^Department of Sociology, The Ohio State University, Columbus, OH, USA; ^3^Preventive Medicine Branch, Walter Reed Army Institute of Research, Silver Spring, MD, USA

**Keywords:** regression, logistic, log-linear, hazard ratio, odds ratio, relative risk, epidemiology, principal component analysis

## Abstract

This paper reviews several common challenges encountered in statistical analyses of epidemiological data for epidemiologists. We focus on the application of linear regression, multivariate logistic regression, and log-linear modeling to epidemiological data. Specific topics include: (a) deletion of outliers, (b) heteroscedasticity in linear regression, (c) limitations of principal component analysis in dimension reduction, (d) hazard ratio vs. odds ratio in a rate comparison analysis, (e) log-linear models with multiple response data, and (f) ordinal logistic vs. multinomial logistic models. As a general rule, a thorough examination of a model’s assumptions against both current data and prior research should precede its use in estimating effects.

## Introduction

Statistics is the study of data collection, organization, abstraction, analysis, interpretation, description, conclusion, and inference (1). It deals with all aspects of data analysis, including planning for data collection, design of studies, model selection, and result interpretation. When analyzing data, an investigator can use descriptive or inferential statistics. One of the main roles of inferential statistics is to make conclusions about a population of interest when data are only available from a sample.

Epidemiology is the study of the distribution and determinants of health-related states or events in specified populations (2, 3). Based on the data from a good sample, epidemiologists can use inferential statistics to make inferences about a cause–effect relationship in the population (4). For epidemiologists, the function of statistics is to determine whether the association observed in a sample actually exists in the population from which the sample is drawn. Statistics is used as a tool to determine whether an association truly exists or it simply occurs by chance (5).

However, choosing the correct study designs and proper models is often challenging for researchers conducting epidemiological studies. The majority of the challenges in statistical inference are actually related to statistical modeling (6). Researchers often rely on statistical software to perform data analyses. Many non-statistician researchers, who do not have strong background in statistics, routinely use popular statistical models provided by the software inappropriately.

Although advanced modeling can be more useful than univariate analyses for detecting and summarizing data patterns, using such models inappropriately may generate a higher risk of bias (7). The assumptions used in most modeling procedures are more restrictive than those used in simple analyses. If one or more of these assumptions are violated, the estimates and tests derived from such modeling may be seriously compromised (8). On many occasions, neither researchers nor the statistical software used carefully check whether all the assumptions required for a given model are valid before a statistical analysis is performed.

This review is not intended to serve as a model selection guide as it is not possible for us to cover every issue in detail. We intend to discuss several issues in data analysis often ignored by non-statisticians. Investigators should make model-selection decisions based on the appropriate data review and the nature of the specific epidemiological question at the time of study design. The more complete answers can be found in many good statistic books. The topics presented are also critical, when we review or reference the published manuscripts.

## Several Common Statistical Issues

### Heteroscedasticity and Outliers in Linear Regression

**Figure 1:**
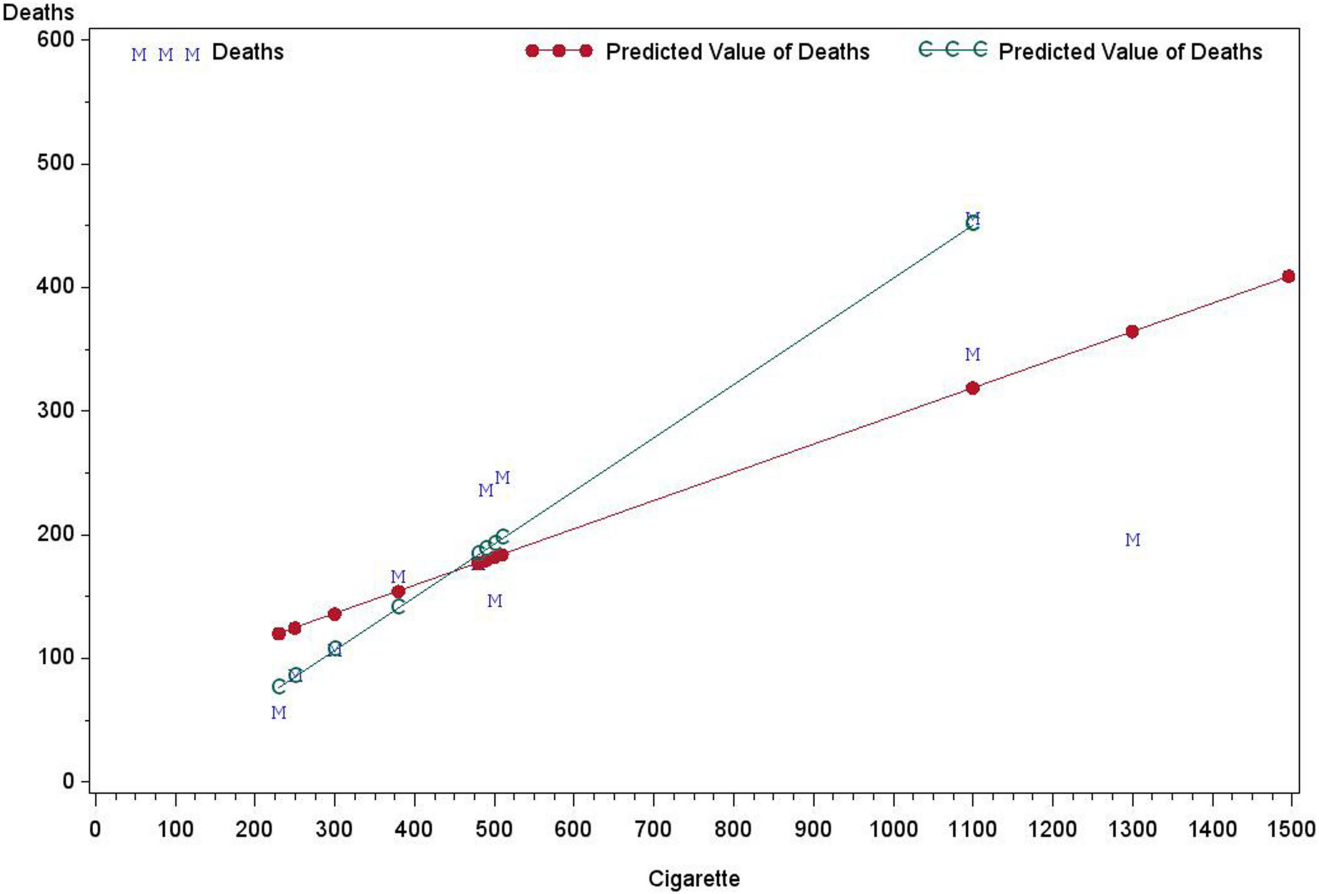
**The deaths by cigarette using: regression with USA and Without USA**.

Heteroscedasticity refers to the circumstance in which the variability of a variable is unequal across the range of values of a second variable that predicts it. As an illustration, Table 1 presents data on the per capita annual consumptions of cigarettes in various countries in 1930 and the death rates (number of deaths per million people) from lung cancer in 1950 in Freedman et al.^1^ As shown in Figure 1, the fitted linear regression lines are different when fitting with and without the United States of America (USA) data.

**Table 1:** **The death rate and cigarette data in Freedman et al**.^1^

Obs	Country	Cigarette	Deaths per million
1	Australia	480	180
2	Canada	500	150
3	Denmark	380	170
4	Finland	1100	350
5	Great Britain	1100	460
6	Iceland	230	60
7	Netherlands	490	240
8	Norway	250	90
9	Sweden	300	110
10	Switzerland	510	250
11	USA	1300	200

Whether to include the USA data in analysis is a question that was originally posted. However, a second, related question must also be answered: should the regression line pass through the original?

This example illustrates some common statistical issues that are often ignored by investigators performing linear regressions. Specifically, there are only 11 data points, so the sample size is too small to make a meaningful conclusion about an association between the death rate and cigarette consumption. Moreover, there are only two independent factors (Country and Cigarette consumption) and one response variable (Death from lung cancer) in the data set. Since there was one observation per country, the country variable cannot be used directly as a predictor. Using cigarette consumption as the only predictor for modeling the death rate is over-simplified. Since death rates from different countries are independent and the errors are approximately normally distributed, those assumptions could be accepted. When performing the linear regression using all 11 records, the USA was considered as an outlier, with its Cook’s *D* = 2.56, Leverage = 0.43, and DFFITS = −4.32, which are commonly used measures of the influence of a data point when performing least squares regression analysis (9). Does being an outlier necessarily support the deletion of the USA data in the analysis? This may seem appropriate as it would yield a better fit with a higher coefficient of determination: *R*^2^ = 0.94 without vs. *R*^2^ = 0.54 with USA data. However, deletion of the USA from the analysis would remove the largest population of consumers (1300) among the 11 countries. Therefore, any conclusion drawn without USA data would be improper. For example, the intercept of the regression without the USA data is 0 and this would imply that the death rate from lung cancer is 0 for non-smoking countries.

**Figure 2:**
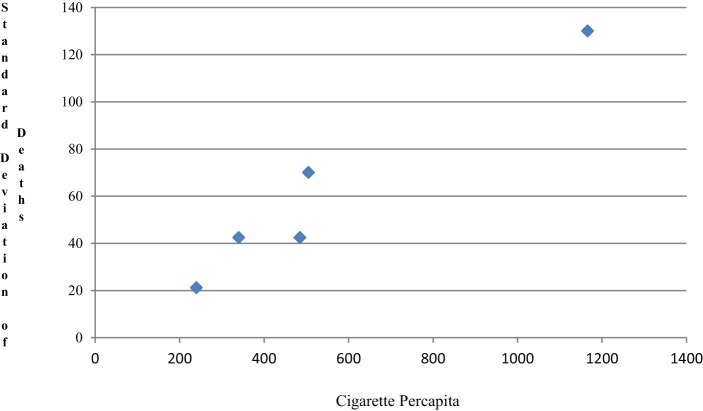
**The SD changes by average cigarette**.

When the 11 countries were categorized by order of cigarette use into five groups (2, 2, 2, 2, 3) respectively, the scatter plot of the SDs of the death rate by average cigarette consumption are shown in Figure 2. Hence, a simple linear model assuming homogeneity of variance does not hold. When linearity or homogeneity of variance is violated, a transformation, such as logarithm, square root, square, and exponential, is often used. If the linearity assumption holds, but the variance is not constant, the dependent variable is usually transformed before performing the regression modeling. If linearity is violated, then either or both of outcome and predictor may be transformed (10).

It can be seen in Figure 1 that points representing higher death rates have larger variances. Two possible transformations of death data could be considered: logarithm and square root. Figure 1 also demonstrates that the variance of deaths is an increasing function of cigarette consumption. Using *Y* to represent death rates and *X* to represent cigarette consumption, the following transformation forms may be considered:

$$\text{Square Root transformation }Y_{1}=\sqrt{Y,}\;V\left(Y_{1}\right)=\frac{1}{4E\left(Y\right)}V\left(Y\right)$$

$$\text{Logarithm transormation }Y_{2}=Ln\left(Y\right),\;V\left(Y_{2}\right)=\frac{1}{E{\left(Y\right)}^{2}}V\left(Y\right)$$

$$\text{Stabilizing variance }Y_{3}=\frac{Y}{X}\;V\left(Y_{3}\right)=\frac{1}{X^{2}}V\left(Y\right)$$

The size for each group is limited for doing two-stage modeling; here, we just used these data to show two-stage modeling as an example. As Figure 2 demonstrates, the relationship between SD of death and cigarette use becomes almost perfectly linear through the origin (by regression, interception = −0.76, *R*^2^ = 0.95); hence, it is easy to show V(*Y*_3_) is constant and the variance is stabilized by using *Y*_3_. Mortality and cigarette use are positively associated. It can be shown that the variance of *Y*_1_ and *Y*_2_ are also stabilized.

We consider the following six regression models varying by transformation of *Y*, order of *X*, and restriction on the error term (ε), α, β’s are corresponding regression coefficients:

Classic Regression Models

Model 1: Y = α + βX + ε
$\text{Model 2: }\sqrt{Y\;}\text{= }\alpha \text{ + }\beta \text{X + }\varepsilon$
Model 3: ln(Y) = α + βX + ε
  Models with non-constant error term
$\text{Model 4: }\frac{Y}{X}\text{ = }\alpha \text{ + }\beta X\text{ + }\varepsilon \text{ then }Y\text{ = }\alpha X\text{ + }\beta X^{\text{2}}\text{ + }X\varepsilon$
$\text{Model 5: }\frac{Y}{X}\text{ = }\alpha \text{ + }\beta \frac{1}{X}\text{ + }\varepsilon \text{ then }Y\text{ = }\alpha X\text{ + }\beta \text{ + }X\varepsilon$
  Two-Stage regression model
$\mathrm{Model}\;6:y_{ij}=\alpha +\beta_{ij}X_{j}+\varepsilon_{ij},\;i=1,2,\ldots ,n_{j},\;j=1,2,\ldots ,5$

Model 1 is the classic regression. The systematic components in Models l and 5 are the same. Models 4 and 5 have a non-constant error term. The variance in Models 4 and 5 is proportional to the square of cigarette consumption. Models 2, 3, and 4 are non-linear regressions. The estimation of regression coefficients in Model 6 is different from other models, as the two-stage regression model is used (10). There are two kinds of errors in Model 6 for the observation:

$$\varepsilon_{ij}=(y_{ij}-\bar{y_{j}})+\left(\bar{y_{j}}-\hat{y_{ij}}\right).$$

The first part is the pure error; the second part is the regression error. After estimating sample SD s_j_ for each group, performing the classic linear regression, and generating the residuals for all records, then define:

(1) $$c_{j}=\frac{s_{j}^{2}}{\frac{1}{n}\sum_{i=1}^{11}e_{i}^{2}}j=1,\ldots ,5$$

using transformation, *Y*′ = *Y*/*c_j_, X*′ = *X*/*c_j_*, adding *C* = 1/*c_j_* as a new predictor to perform the multivariate analysis. Using the cigarette consumption and death rate data described earlier, the corresponding regression coefficient parameter estimates for Models 1–6 generated by SAS 9.3 are listed in Table 2.

**Table 2:** **Parameter estimation and model fitting**.

Model	Parameter	Estimate	SE	*T* value	*p* value	*R*^2^
1	α	67.561	49.06	1.38	0.2	0.54
	β	0.228	0.07	3.27	0.01	
2	α	9.074	1.644	5.52	<0.0001	0.56
	β	0.008	0.002	3.36	0.01	
3	α	4.483	0.248	18.07	<0.0001	0.54
	β	0.001	0	3.25	0.01	
4	α	0.422	0.058	7.24	<0.0001	0.26
	β	0	0	−1.2	0.26	
5	α	0.352	0.071	4.98	<0.0001	0.37
	β	4.274	27.749	0.15	0.88	
6	α	−44.763	58.595	−0.76	0.467	0.73
	c	39.883	33.856	1.18	0.273	
	β	0.366	0.123	2.98	0.018	

In Model 5, the intercept α = 0.352 is the estimated linear effect (slope) of cigarette use on death rate, which should be compared to the slope β = 0.228 with the USA data and β = 0.430 (not shown in table) without the USA data in Model 1. When the USA data are included, the slope is higher when using Model 5 than when using Model 1. However, the SE remains the same (0.07). If using Model 5 for both data sets with and without the USA data, the difference between the two regression lines [slope (without the USA data) = 0.438, slope (with USA data) = 0.352] is much smaller than that when using Model 1. After controlling for the heteroscedasticity, the outlier effect from the USA data is reduced (Cook’s *D* = 0.76, Leverage = 0.24 and DFFITS = −1.68). Model 6 generates a similar estimate of β = 0.366 (β = 0.414 without the USA data) with a higher SE of 0.123. However, the model fits the data best with a *R*^2^ of 0.73. The effect difference between including and excluding the USA data is reduced. In conclusion, both Models 5 and 6 might be better regression models for fitting the data with heteroscedasticity.

### Collinearity and Principal Component Analysis

The specific conclusion on the effect of individual factors from a multiple regression equation depends on whether the predictor is correlated with other predictors (11). The coefficient of a predictor measures the change of the response variable when the predictor changes by one unit, while other predictors are held constant. When one or more predictors are removed from the equation, the coefficient of the remaining predictors should not change. However, in practice, the interpretation may not be valid because predictors are often correlated making it difficult to change one, while holding the others constant. In addition, if a strong association exists among predictors, referred to as collinearity, the interpretation of regression coefficients becomes unreliable. When collinearity exists, even minor changes, such as removing or adding a predictor or deleting a few records, may lead to unpredictable changes in the estimated coefficients, or even changes in sign or an increase in the estimated SEs.

Table 3 shows Hald’s data with a new response variable (*U*) generated by Hadi [(10), Chapter 6]. The estimated regression coefficients on *x*1, *x*4, *x*1–*x*3, and *x*1–*x*4 are provided in Table 4. For univariate models, such as Models 1 and 2, both *X*_1_ and *X*_4_ are not statistically significant (*p* < 0.05), the regression coefficient is close to 0 (0.05 and 0.26, respectively), and the model fits poorly. Similar results are found for *X*_2_ and *X*_3_ when using a univariate model. When *U* is regressed on *X*_1_, *X*_2_, and *X*_3_ (Model 3), the regression coefficients do not change much, but the SE for *X*_1_ is doubled. In Model 4, when *X*_4_ is added into Model 3, all regression coefficients increase more than 20 times in magnitude, and the SE also increases dramatically. It can be shown that high collinearity exists among *X*_1_, *X*_2_, *X*_3_, and *X*_4_, and there is a strong association between the response and these highly correlated variables. However, it becomes difficult or impossible to distinguish their individual influences on the response variable (impossible to come up with reliable estimates of their individual regression coefficients). This poses a real problem if the purpose of the study is to estimate the contributions of individual predictors.

**Table 3:** **Hald’s data**.

*U*	*x*1	*x*2	*x*3	*x*4
0.955	7	26	6	60
0.746	1	29	15	52
−2.323	11	56	8	20
−0.82	11	31	8	47
0.471	7	52	6	33
−0.299	11	55	9	22
0.21	3	71	17	6
0.558	1	31	22	44
−0.119	2	54	18	22
0.496	21	47	4	26
0.781	1	40	23	34
0.918	11	66	9	12
0.918	10	68	8	12

**Table 4:** **Effects of collinearity**.

Model	Variable	DF	Parameter	SE	Pr > |*t*|	*R*^2^
1	Intercept	1	4.49	4.33	0.3227	0.05
	*X*_1_	1	−0.34	0.46	0.4724	
2	Intercept	1	−0.76	5.60	0.8948	0.026
	*X*_4_	1	0.09	0.16	0.5988	
3	Intercept	1	6.81	17.62	0.708	0.05
	*X*_1_	1	−0.37	0.92	0.6962	
	*X*_2_	1	−0.03	0.20	0.8775	
	*X*_3_	1	−0.05	0.83	0.9522	
4	Intercept	1	−804.74	132.13	0.0003	0.83
	*X*_1_	1	7.90	1.40	0.0005	
	*X*_2_	1	8.35	1.36	0.0003	
	*X*_3_	1	8.41	1.42	0.0004	
	*X*_4_	1	8.23	1.34	0.0003	

Principal component analysis (PCA) is one of the most commonly used approaches to resolving collinearity and reducing the dimensions for high dimensional data analysis. PCA uses an orthogonal transformation to convert a set of observations of potentially correlated variables into a set of values of linearly uncorrelated variables (12). The number of principal components used is often fewer than the number of original variables (13). The first principal component has the largest variance, followed by the subsequent components in decreasing order by their associated variances under the constraint of orthogonal transformation to the preceding components. PCA might offer users with lower-dimensional components as the predictors in the linear regression. Then, the dimensionality of the transformed data is reduced to achieve similar model fitting. Whether this is true and how many principal components should be used are common questions. Unfortunately, PCA does not imply that the first component is the most significant on the outcome. Neither does PCA guarantee that only a few principal components can fit the model well. For Hald’s data (10), the eigenvalues and eigenvectors from PCA are listed in Table 5. The 4th eigenvalue is almost 0, which means the linear combination 0.241*x1 + 0.642*x2 + 0.268*x3 + 0.677*x4 is near 0 (0.002).

**Table 5:** **Eigenvalue and eigenvactor for Hald’s data**.

	Prin1	Prin2	Prin3	Prin4
Eigenvalue	2.236	1.576	0.187	0.002
*x*1	0.476	−0.509	0.676	0.241
*x*2	0.564	0.414	−0.314	0.642
*x*3	−0.394	0.605	0.638	0.268
*x*4	−0.548	−0.451	−0.195	0.677

The regression analyses on (prin1, prin2, prin3) and (prin1, prin2, prin3, prin4) were performed, respectively, and the results are shown in Table 6. When the first three principal components are used as predictors, it can be seen that none of the first three principal components are significant (*p* < 0.05), and the coefficient of determination (*R*-square) is only approximately 0.06, When all four principal components are included in the model, the only significant principal component is “prin4,” with almost 0 variance. The *R*-square is 0.83. This example illustrates that principal components with high variances do not necessarily have large effects on the response outcome. Moreover, the number of orthogonal PCA components used to replace the high dimensional original predictors in the modeling cannot be decided in general.

**Table 6:** **Regression analysis by PCA score**.

Model	Variable	Parameter	SE	Pr > |*t*|	*R*^2^
Prin1–prin3	Intercept	0.19	0.29	0.52	0.0596
	Prin1	−0.13	0.2	0.53	
	Prin2	0.06	0.24	0.82	
	Prin3	−0.2	0.69	0.77	
Prin1–prin4	Intercept	0.19	0.13	0.17	0.8345
	Prin1	−0.13	0.09	0.18	
	Prin2	0.06	0.11	0.61	
	Prin3	−0.2	0.31	0.53	
	Prin4	20.22	3.3	0.0003	

### Interpreting Regression Output

The regression analysis generates an equation that allows predicting values of a dependent variable through the values of one or more independent variables. The equation is written as:

(2) $$Y_{i}=\alpha +\beta_{1}X_{1i}+\beta_{2}X_{2i}+\ldots +\beta_{p}X_{pi}+\varepsilon_{i},i=1,2,\ldots ,n$$

where *Y* is the dependent variable, and *X*_1_, *X*_2_, …, *X*_p_ are the independent variables used to predict *Y*. The coefficients β_1_, β_2_, … describe the size of the effects of the independent variables on the dependent variable *Y*, and α (also known as the intercept) is the predicted value of *Y* when all the independent variables are equal to 0. Holding all *X*_2_ to *X*_p_ constant, when *X*_1_ increases one unit, *Y* will increase β_1_ units, β_2_, β_3_, etc., follow the similar interpretation. If the assumption of independence among all *X_i_* is true, the interpretation might be correct. However, in practice, the independent assumption of the predictors is often violated and the meaning of the coefficients changes. Suppose there are two predictors, *X*_1_ and *X*_2_, then the actual meaning of β_2_ is the regression effect on two residuals: the residuals of *X*_2_ regressing on *X*_1_ as the predictor, and the residuals of *Y* regressing on *X*_1_ as the outcome. For multiple regressions, the adjusted regression coefficient from a newly added predictor *X*_k_ is estimated by two residuals from two regression models with the same predictors of *X*_1_ to *X*_k−1_ with different outcomes of *Y* and X_k_.

As an illustration, the following data set, showing in SAS coding, has six predictors (*X*_1_ through *X*_6_) and one outcome of *Y*, but only *X*_1_, *X*_2_, and *X*_3_ are used to set up the following four models:

Model 1: *Y* = α_1_ + β_11_*X*_1_ + β_12_*X*_2_, the residual variable denoted as *r*_*y*_*x*1*x*2
Model 2: *X*_3_ = α_2_ + β_21_*X*_1_ + β_22_*X*_2_, the residual variable is *r*_*x*3_*x*1*x*2
Model 3: *Y* = α_1_ + β_1_*X*_1_ + β_2_*X*_2_ + β_3_*X*_3_
Model 4: *r*_*y*_*x*1*x*2 = α_0_ + β_3_′ *r*_*x*3_*x*1*x*2.

**data** reg;
 input *x*1–*x*6 *y* @@;
id = _N_;
datalines;
44 89.47 44.609 11.37 62 178 182 40 75.07 45.313 10.07 62 185 185
44 85.84 54.297 8.65 45 156 168 42 68.15 59.571 8.17 40 166 172
38 89.02 49.874 9.22 55 178 180 47 77.45 44.811 11.63 58 176 176
40 75.98 45.681 11.95 70 176 180 43 81.19 49.091 10.85 64 162 170
44 81.42 39.442 13.08 63 174 176 38 81.87 60.055 8.63 48 170 186
44 73.03 50.541 10.13 45 168 168 45 87.66 37.388 14.03 56 186 192
45 66.45 44.754 11.12 51 176 176 47 79.15 47.273 10.60 47 162 164
;

Model 3 is a multiple linear regression model and Model 4 is a simple linear regression on two residuals obtained from Model 1 and Model 2. Table 7 shows the effect of predictor *X*_3_ in Model 3 and the effect of its residual in Model 4.

**Table 7:** **Comparison of regression coefficients in multiple regressions**.

Model	Variable	Parameter	SE	*p* value
3	Intercept	278.916	48.804	0.000
	*x*1	−1.826	0.685	0.024
	*x*2	0.132	0.254	0.614
	*x*3	−0.712	0.312	0.046
4	Intercept	0.000	1.544	1.000
	*r*_*x*3_*x*1*x*2	−0.712	0.285	0.028

**Figure 3:**
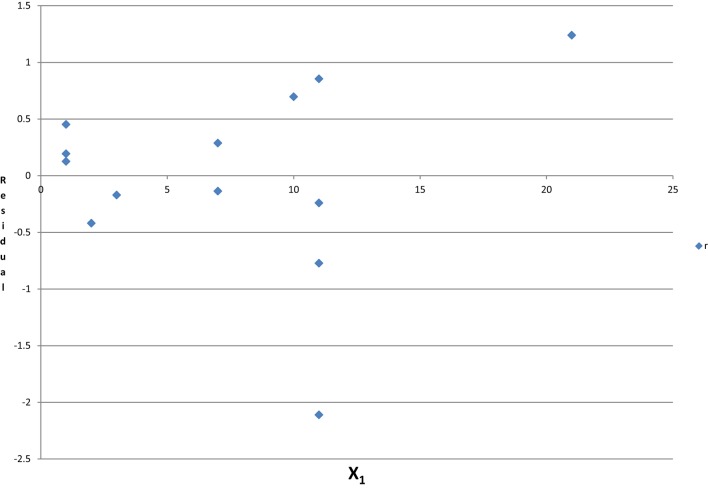
**The unexplained information of *X*_1_ and *X*_1_ by controlling for *X*_2_, *X*_3_, and *X*_4_**.

The regression coefficient of β_3_ = −0.712 for *X*_3_ on *Y* in Model 3 is the same as the regression coefficient of β_3_′ = −0.712 in Model 4 using two residuals: *r*_*x*3_*x*1*x*2 on *r*_*y*_*x*1*x*2. In a multiple linear regression, the regression coefficient of a predictor is the effect of the unexplained information of that predictor by other predictors on the unexplained information of the outcome by the same group of predictors. If a predictor can be predicted by other predictors, i.e., they are correlated; the residuals (unexplained information) would be random and have no relation to that predictor. The adjusted effect of that factor in the model including all of the correlated independent predictors is unreliable, and the sign and the magnitude may be random. Based on Hald’s data in Table 3, the adjusted regression coefficient of *X*_1_, the unexplained information (residual) after controlling *X*_2_, *X*_3_ and *X*_4_, is independent of *X*_1_ (see Figure 3). Those results support that, if collinearity exists, the adjusted effect of *X*_1_ and the unadjusted effect are unrelated, and the effect changes unpredictably.

### Categorical Data Analysis: Logistic Regression or Log-Linear Regression

In epidemiology, logistic regression is well known and commonly used. Logistic regression is a special case of the generalized linear model and thus analogous to linear regression (14). Logistic regression is often used in the analysis of binary response data based on one or more predictor variables, where odds ratios (ORs) are often reported. If the response has multiple qualitative response levels, the logistic model becomes more complex and involves multiple outcome functions. If all predictors are categorical variables, a log-linear model may be used.

Similar to logistic regression, log-linear model is also a special case of generalized linear model (15). Log-linear models analyze contingency table data by taking the natural logarithm of the cell frequencies. In many cases, logistic and log-linear models are equivalent to one another (16). All variables investigated by a log-linear model are treated as response variables and not distinguished as dependent or independent as in the analysis of binary response data in a logistic regression.

The log-linear model is more flexible and demonstrates associations among two or more variables, by constructing a multi-way contingency table. In general, if only one variable is treated as the outcome (response), and all others are predictors, it is more convenient to use a logistic regression. If there are more than one response variable, a log-linear model should be used.

This distinction is illustrated in the following analysis of results from an employee survey (Table 8). “Grade” represents a four-point job satisfaction scale, with *A* being the highest and *D* the lowest. “Load” represents a working pressure scale, <4, 4, 5, and >5. Two other variables are age (<25, ≥25) and “Graduate” (whether the respondent graduated from college). Both log-linear and logistic models are used with two possible outcomes: Grade and Load.

**Table 8:** **Employee survey data**.

Obs	Age	Graduate	Load	Grade			
				A	B	C	D
1	<25	Yes	<4	10	7	9	8
2	<25	No	<4	18	28	15	12
3	>25	Yes	<4	12	13	11	9
4	>25	No	<4	38	28	22	14
5	<25	Yes	4	17	23	32	19
6	<25	No	4	25	27	21	11
7	≥25	Yes	4	12	24	37	27
8	≥25	No	4	17	29	31	9
9	≤25	Yes	5	7	6	12	15
10	≤25	No	5	11	25	34	21
11	≥25	Yes	5	6	12	9	44
12	≥25	No	5	12	39	20	22
13	≤25	Yes	>5	9	9	13	18
14	≤25	No	>5	12	24	29	23
15	≥25	Yes	>5	8	9	6	40
16	≥25	No	>5	15	29	23	24

Starting from a full model with a backward selection, the simplest and the most acceptable model is (grade|age|graduate, grade|load, load|graduate), where *A*|*B*|*C* = A B A*B C A*C B*C A*B*C, A*B is the interaction of the two factors *A* and *B*, and A*B*C is the three-way interaction among *A, B*, and *C*. The selected model contains one three-way interaction, five two-way interactions, and four main effects. When grade is used as the only response in the logistic model, the association of load and graduate cannot be estimated, because load*graduate cannot be included in the model. When load is used as the unique response in a logistic regression, the associations among grade, age, and graduate cannot be estimated. There is no equivalent logistic model for this circumstance, in which a log-linear model would be appropriate. Table 9 compares the results from two regression models. One is the logistic model by using grade as the outcome. The other is the log-linear model, which is equivalent or the most closely related to the selected logistic model.

**Table 9:** **Comparison of the estimates from log-linear and logistic models**.

Log-linear model				Logistic model			
Source	DF	Chi-Square	Pr > ChiSq	Effect	DF	Wald Chi-square	Pr > ChiSq
Grade	3	21.72	<0.0001				
Age	1	5.77	0.02				
Age*grade	3	6.96	0.07	Age	3	7.05	0.07
Graduate	1	53.63	<0.0001				
Graduate*grade	3	48.76	<0.0001	Graduate	3	47.98	<0.0001
Age*graduate	1	0.59	0.44				
Age*graduate*grade	3	8.64	0.03	Age*graduate	3	6.90	0.08
Load	3	24.46	<0.0001				
Load*grade	9	79.55	<0.0001	Load	9	74.74	<0.0001
Load*graduate	3	40.73	<0.0001				

The SAS codes are:

proc logistic data = table1 rorder = data;
freq count;
class Load age graduate/param = glm;
model grade = age|graduate load/link = glogit;
title 'Logistic model: baseline';
run;

proc genmod data = table1 rorder = data;
class load age graduate grade;
model count = grade|age|graduate grade|load load|graduate/type3 dist = poi link = log;
title 'Reduced log-linear model';
run;

It can be seen that for the associations of grade with age or graduate, the estimation results from two models are similar. However, there are some differences between the two models in evaluating the association of grade with the interaction of age*graduate. First, the parameter estimate is significant by using the log-linear model (*p* = 0.03), while it is weakly significant by using the logistic model (*p* = 0.08). The grade score changes by age and graduate as well as from their interaction. In other words, the distribution of grade score by age is significantly different for those who have an advanced degree from those who do not, which was found by using log-linear modeling, but it was not found if using logistic model. In addition, the log-linear model can examine the associations between load and graduate and age and graduate, while the logistic cannot do that. Therefore, the log-linear model outperforms the logistic model, as it can better explain the associations among the four factors in the contingency table. If more than one response variable are of interest to investigators, the log-linear model should be used rather than logistic model.

### Multinomial and Ordinal Logistic Regression

Logistic regression is commonly used when the outcome is categorical. By using the natural log of the odds of the outcome as the dependent variable, we usually examine the odds of an outcome occurring and the relationship with other factors similar to multiple linear regression (16).

When the outcome is not dichotomous, the outcome measurement type should be distinguished first; a step often ignored by some investigators. There are two kinds of logistic modeling: multinomial logistic regression handles the case of a multi-way categorical dependent variable (17) and ordinal logistic regression handles an ordinal dependent variable (18).

#### Multinomial Logistic Model

The dataset, in the following example from SAS, contains the results of hypothetical users testing three brands of computer games. Users rated the games on a five-point scale from very good (vg) to very bad (vb). The analysis is performed to estimate the differences in the ratings of the three games. The variable score represents the game rating scales, and the variable game represents the games tested. The variable count reflects the number of testers in the underlying rating category for the underlying game. The multinomial or baseline logistic model used to define the regression functions is written as:

(3) $$\ln\left(\frac{p_{ijk}}{p_{i5k}}\right)=\alpha_{j}+\beta_{ij}\;{\text{game}}_{ijk}+\varepsilon_{ij}\;i=1,2,j=1,2,3,4,k=1,2,\ldots ,n$$

There are four outcome functions for the log odds compared with the very bad rating group (reference group): ln(*p*(vg)/*p*(vb)), ln(*p*(g)/*p*(vb)), ln(*p*(m)/*p*(vb)), ln(*p*(b)/*p*(vb)).

The SAS codes are:

Data Compgame;
input count game$ score$ @@;
datalines;
70 game1 vg 71 game1 g 151 game1 m 60 game1 b 46 game1 vb
20 game2 vg 36 game2 g 130 game2 m 80 game2 b 70 game2 vb
50 game3 vg 55 game3 g 140 game3 m 72 game3 b 50 game3 vb
;
Proc logistic data = Compgame rorder = data;/*rorder function keeps the outcome functions as the order as the order in data: vg, g, m, b, vb*/
freq count;
class game/param = glm;
model score = game/link = glogit;
run;

Table 10 summarizes the model fitting and estimation results. Below are some interpretations of the results:

1. There are four intercepts for the four baseline logit outcomes, with the first for the first logit: ln(*p*(vg)/*p*(vb)), which is −1.25E−13 = 0. It is the log odds for Game 3: for Game 3, vg = vb = 50, hence ln(*p*_1_/*p*_5_) = 0. Similar to the intercept1, all intercepts are the log odds relative to *p*_5_ for Game3.
2. There are eight regression coefficients β’s, which are the differences of the four logits between Game 1 and Game 3 and between Game 2 and Game 3. The first β of Game 1 is 0.42 for level of vg, which is logit difference between Game 1 and Game 3 for the response function ln(*p*(vg)/*p*(vb)). It is positive, meaning that the rating of Game 1 is better than that of Game 3, with more ratings of “very good” received for Game 1. Exponentiation this value exp(0.4199) = 1.522 gives the OR between Game 1 and Game 3 for comparing the very good score with very bad score. The percentage ratio of rating GAME 1 to be very good vs. rating GAME 1 to be very bad is roughly 52% higher than the percentage ratio for GAME 3. However, the difference is not statistically significant as the *p*-value is only about 0.13.
3. The interpretations are similar for others, for Game 2, the percentage ratio receiving scores vg vs. vb is significantly lower than for Game 3, with an OR of 0.286. The number of very good ratings for Game 2 is much lower than that for Game 3.

**Table 10:** **Results from multinomial logistic regression**.^a^

Parameter	Score	Estimate (β)	SE	*p* value	Model fitting		
Intercept	vg	0.000	0.200	1.000	AIC	3356	3323
Intercept	g	0.095	0.195	0.626	SC	3376	3383
Intercept	m	1.030	0.165	<0.0001	−2Log L	3348	3299
Intercept	b	0.365	0.184	0.048	OR^a^	95% CI	
Game 1	vg	0.420	0.276	0.128	1.522	0.886	2.612
Game 1	g	0.339	0.272	0.213	1.403	0.823	2.391
Game 1	m	0.159	0.236	0.500	1.172	0.739	1.86
Game 1	b	−0.099	0.269	0.713	0.906	0.535	1.534
Game 2	vg	−1.253	0.323	0.000	0.286	0.152	0.538
Game 2	g	−0.760	0.283	0.007	0.468	0.268	0.815
Game 2	m	−0.411	0.222	0.064	0.663	0.43	1.024
Game 2	b	−0.231	0.246	0.348	0.794	0.490	1.286

*^a^The Reference group is game 3 and score = vb*.

If using the data with score = vg and score = vb only, the score becomes a binary outcome. The function is log(*p*_s_(vg|(under vg and vb)))/(1 − *p*_s_(vg|(under vg and vb))) = log(*p*(vg)/*p*(vb)). Using the binary outcome logistic model generates the same results as those using the multinomial logistic regression for the response function for that subset of the data. The parameter estimate for Game 1 is 0.4199 and the OR for the two ratings *p*(vg) to *p*(vb) is 1.52. Hence, for the data above, without other control factors involved in the model, the multinomial model simply combines the dichotomous logistic regressions together. However, if there are other covariates, the model will be more complex. Usually, when the regressors include both categorical and continuous predictors, there are four different model designs that can be employed: (1) same intercept, same slope; (2) different intercepts same slope; (3) different intercepts, different slopes; (4) same intercept; different slopes. If design 3 – different intercepts and different slopes – is used, the combined multinomial logistic model and the binary logistic model by using the same reference level will generate the same regression results.

#### Ordinal Logistic Model

Ordinal (or ordered) logistic model can be used for ordinal dependent variables. Since the score measurement is clearly ordered, assuming *p*_1_, *p*_2_, *p*_3_, *p*_4_, and *p*_5_ are the probabilities to be tested, scored as vg, g, m, b, and vb, the cumulative logistic functions are proportional and can be defined as follows:

**Table:** 

Response function	Assigned score
Very good: ln(*p*_1_/(*p*_2_ + *p*_3_ + *p*_4_ + *p*_5_))	0
Good: ln(*p*_1_ + *p*_2_)/(*p*_3_ + *p*_4_ + *p*_5_)	1
Medium: ln(*p*_1_ + *p*_2_ + *p*_3_)/(*p*_4_ + *p*_5_)	2
Bad: ln((*p*_1_ + *p*_2_ + *p*_3_ + *p*_4_)/*p*_5_)	3

The regression equation:

(4) $$\ln\left(\frac{p_{ijk}}{p_{i5k}}\right)=\alpha_{j}+\beta_{i}{\text{ game}}_{ijk}+\varepsilon_{ijk}\;i=1,2,j=1,2,3,4,k=1,2,\ldots ,n$$

The SAS codes are:

proc logistic data = Compgame rorder = data;/* rorder function assigns the order of the four outcome functions as the order of the data, the scores are 0, 1, 2 and 3*/
freq count;
class game/param = glm;
model score = game/link = clogit;/*clogit performing the ordinal logistic regression, assuming the increasing rate or the four lgits is constant*/
run;

Table 11 shows that assuming the odds to be proportional, the option of “clogit” uses the cumulative odds to describe the game effects on the assigned score. The four cumulative logits are assumed to have the same “distance,” ranged from 0 to 3 on the cumulative logit function. The slope estimation for a game is the relative change of the logarithm, of cumulative odds, comparing with Game 3. For Game 1, the slope is 0.3098, it implies the counts are non-even comparing with Game 3, the logarithm of cumulative OR is 0.3098, which is the change from one category to another category in sequential with the reference level of vb. Hence, there are more good readings for Game 1 as compared with Game 3. For Game 2, the slope is −0.5748. Hence, there are more bad readings for Game 2 as compared with Game 3.

**Table 11:** **Analysis of maximum likelihood estimates**.

Parameter		DF	Estimate (slope)	SE	Wald Chi-Square	Pr > ChiSq
Intercept	vg	1	−1.9087	0.1189	257.592	<0.0001
Intercept	g	1	−0.9356	0.1022	83.7943	<0.0001
Intercept	m	1	0.7305	0.1004	52.9434	<0.0001
Intercept	b	1	1.8493	0.1162	253.437	<0.0001
Game	Game 1	1	0.3098	0.1307	5.6179	0.0178
Game	Game 2	1	−0.5748	0.1365	17.7412	<0.0001
Game	Game 3	0	0	–	–	

### Relative Risk, Odds Ratio, and Hazard Ratio

Odds ratio is one of three main ways to quantify how strongly the presence or absence of property *A* is associated with the presence or absence of property *B* in a given population (19). OR can be readily obtained from logistic regression. On the other hand, hazard ratio (HR) is the ratio of the hazard rates corresponding to the conditions described by two levels of an explanatory variable and is often presented in a survival analysis. Notably, HR is often treated as a ratio of death probability (20). In fact, both OR and HR can be used in generalized linear regression to approximate relative risk (RR); a fact many researchers may not appreciate. HR is not OR in concept, and HR is popular in survival analysis. However, it is less known to researchers that HR can be used to compare rates as a ratio measure, especially for a matched case–control study. Under certain conditions, while both OR and HR are good estimates of RR, HR is better than OR in approximating RR.

In the following example, *p*_1_ is the prevalence of a disease (say lung cancer) among an exposure group (say smoking) and let *p*_2_ be the prevalence of the same disease among an unexposed group, then RR = *p*_1_/*p*_2_ is the RR of disease; that is the probability of disease in the exposed group compared with the probability of the disease in the unexposed (comparison) group. When the disease (e.g., lung cancer) is a rare event, it can be extremely inefficient to study cancer in relation to certain exposures in a cohort study. As an alternative, a case–control study design is often used. For case–control studies, it is impossible to estimate the prevalence of *p*_1_ and *p*_2_ from the selected sample with given number of cases and controls. Hence, we can only use ORs to estimate the associations in such studies. The log odds or logit, ln(*p*/(1 − *p*)), is used as the outcome in logistic regression. The simple logistic regression can be written as:

(5) $$\ln\left(\frac{p_{i}}{1-p_{i}}\right)=\alpha +\beta X_{i}+\varepsilon_{i}$$

For the continuous factor *X*, each unit of increase in *X* will lead to β units of increase in logit. For categorical factor, say *X* = 0, refers to the non-exposure group and *X* = 1 refers to the exposure group, the natural logarithm of the OR is equal to β, hence

(6) $$\text{OR}=\frac{p_{1}\left(1-p_{0}\right)}{p_{0}\left(1-p_{1}\right)}=\exp\left(\beta \right)$$

where *p*_1_ = *p*(*Y* = 1|*x* = 1), *p*_0_ = *p*(*Y* = 1|*x* = 0).

However, logit link is not the only function that can be used in generalized linear regression. If choosing log–log link, the corresponding generalized regression is

(7) $$\ln\left(-\ln\left(1-p_{i}\right)\right)=\alpha +\beta X_{i}+\varepsilon_{i}$$

with the same predictor of *X*. Using the same prevalence notation, for one unit increasing of *X*, the log–log non-event probability changes β units, i.e.,

(8) $$\ln\left(-\ln\left(1-p_{1}\right)\right)-\ln\left(-\ln\left(1-p_{0}\right)\right)=\beta$$

then,

(9) $$\left(1-p_{1}\right)={\left(1-p_{0}\right)}^{\text{exp}\left(\beta \right)}={\left(1-p_{0}\right)}^{\text{HR}}$$

where HR = exp(β). For a binary response, *p*_1_ is the prevalence of disease in the exposed group. Thus, (1 − *p*_1_) is the probability of no disease (non-event, or survival from disease) in the exposed group. Similarly, (1 − *p*_0_) is the probability of no disease (non-event) in the unexposed group.

We can also extend these analyses for ordinal outcomes. Suppose we have an ordinal variable *Z* with multiple levels of responses, e.g., different healthy levels: *Z* = 0: seriously sick, *Z* = 1: moderately sick, *Z* = 2: lightly sick, and *Z* = 3: healthy. Let’s consider these values as survival time. Using the cumulative probabilities, we can apply the cumulative logistic regression with the log–log link function as below:

$$\ln\left(\frac{p\left(Z_{i}\leq z_{0}\right)}{p\left(Z_{i}>z_{0}\right)}\right)=\alpha +\beta X_{i}+\varepsilon_{i},z_{0}=0,1,2,3$$

The log–log link regression is

(10) $$\ln\left(-\ln\left(p\left(Z_{i}>z_{0}\right)\right)\right)=\alpha +\beta X_{i}+\varepsilon_{i},z_{0}=0,1,2,3$$

On the other hand, the proportional hazard (PH) model estimating HR may also be applied. The PH model is popular in survival analysis. It can be used to perform conditional logistic analysis, and has been used for matched case–control studies. If the true prevalence of a disease is low, and the true RR is not too high, as shown below, both the OR and HR are approximately equal to RR, i.e.,

$$\text{OR}=\frac{p_{1}\left(1-p_{0}\right)}{p_{0}\left(1-p_{1}\right)}\approx \frac{p_{1}}{p_{0}}=\text{RR}$$

$$\left(1-p_{1}\right)={\left(1-p_{0}\right)}^{\text{HR}}\approx 1-\text{HR*}p_{0},\text{then HR}\approx \text{RR}$$

The following numerical example was generated for RR = 1.1, 1.5, 2.0, 3.0, and 4.0 with different *p*_0_ from 0.01 to 0.2. Both ORs and HRs were calculated. It can be seen from Table 12, for a RR of 3 or higher and the prevalence of no disease >0.2, that while both OR and HR perform poorly in estimating RR, yet HR is always better.

**Table 12:** **Comparisons between OR and HR for approximation of the RR**.

RR	*p*_1_	*p*_0_	OR	HR
1.1	0.011	0.01	1.1011	1.1006
	0.11	0.1	1.112	1.106
	0.22	0.2	1.128	1.113
1.5	0.015	0.01	1.508	1.504
	0.15	0.1	1.588	1.543
	0.3	0.2	1.714	1.598
2.0	0.02	0.01	2.020	2.010
	0.11	0.1	1.112	1.106
	0.22	0.2	1.128	1.113
3.0	0.03	0.01	3.062	3.031
	0.3	0.1	3.857	3.385
	0.6	0.2	6.000	4.106
4.0	0.4	0.1	6.000	4.848
	0.4	0.1	6.000	4.848
	0.8	0.2	16.000	7.213

## Discussions

Setting up an appropriate research question or hypothesis is the foundation of a scientific research study. Selecting proper methodology and study design are essential for the study results to be interpretable and to have clinical relevance. Different study designs may have particular methodological issues and constraints; a common challenge is to avoid potential biases. The potential biases, while often unavoidable and inherent to certain study designs, can limit the relevance and applicability of a given study. It is important to address the potential biases early in the design phase, so as to ensure an appropriate design for the hypothesis and to outline procedures for data collection and analysis (21). While some biases can be adjusted or corrected in the statistical analysis, many others cannot and may render the results of a study invalid. Investigators must be aware of the presence of bias, its effect on validity, and how it can lead to data misinterpretation and limit the applicability or generalizability of a given study [Hartling et al. (22), Agency for Healthcare Research and Quality (USA) (23)].

In this manuscript, several statistical modeling techniques have been reviewed, which often challenge non-statistician investigators. As pointed out earlier, the quality of the findings derived from statistical analyses depends on the model assumptions and data quality. In summary, the examples and discussions in this manuscript show that: (1) When any non-independent predictor is included in a model, the estimation of the exposure effect will vary from its true effect; (2) One can obtain an effect estimate of acceptable precision only by making certain assumptions that the effects of all the covariates are independent of each other and follow a linear response curve; (3) If these assumptions are incorrect, the estimates are likely to be biased; (4) If more than one response factor is involved in contingency table analysis, a log-linear model might offer a better estimate than a logistic regression model; (5) The HR can be used to approximate RR; (6) log–log linear model could be applied to do the data analysis as the logistic regression does. HR might be better than OR in the estimation of RR.

In conclusion, when conducting statistical analyses, one should always evaluate the study hypothesis; check the data type and data quality; check the underlying assumptions, and finally, carefully interpret and present the results. Because some statistical techniques need users to have a strong statistical background, software developers may consider improving the existing statistical packages by including information for non-statistician users to check the data quality and model assumptions automatically, perform suitable statistical analyses, and present appropriate interpretations.

## Author Contributions

YL provided ideas and drafted of the manuscript. All other authors participated revising and polishing the manuscript.

## Conflict of Interest Statement

The research was conducted in the absence of any commercial or financial relationships that could be construed as a potential conflict of interest. Material has been reviewed by the Walter Reed Army Institute of Research. There is no objection to its presentation and/or publication. The opinions or assertions contained herein are the private views of the author, and are not to be construed as official, or as reflecting true views of the Department of the Army, the Department of Defense or Food and Drug Administration (FDA). The reviewer CT and handling Editor declared their shared affiliation, and the handling Editor states that the process nevertheless met the standards of a fair and objective review.

## Footnotes

^1^Data set in Freedman, et al. *Outliers, Leverage & Influential Points in Regression* (1991). Available from: http://math.etsu.edu/1530/Outliers.doc
